# Evolutionary Algorithm Based Feature Optimization for Multi-Channel EEG Classification

**DOI:** 10.3389/fnins.2017.00028

**Published:** 2017-02-01

**Authors:** Yubo Wang, Kalyana C. Veluvolu

**Affiliations:** ^1^School of Life Science and Technology, Xidian UniversityXi'an, China; ^2^School of Electronics Engineering, College of IT Engineering, Kyungpook National UniversityDaegu, South Korea

**Keywords:** BCI, evolutionary algorithm, Fourier linear combiner, feature optimization

## Abstract

The most BCI systems that rely on EEG signals employ Fourier based methods for time-frequency decomposition for feature extraction. The band-limited multiple Fourier linear combiner is well-suited for such band-limited signals due to its real-time applicability. Despite the improved performance of these techniques in two channel settings, its application in multiple-channel EEG is not straightforward and challenging. As more channels are available, a spatial filter will be required to eliminate the noise and preserve the required useful information. Moreover, multiple-channel EEG also adds the high dimensionality to the frequency feature space. Feature selection will be required to stabilize the performance of the classifier. In this paper, we develop a new method based on Evolutionary Algorithm (EA) to solve these two problems simultaneously. The real-valued EA encodes both the spatial filter estimates and the feature selection into its solution and optimizes it with respect to the classification error. Three Fourier based designs are tested in this paper. Our results show that the combination of Fourier based method with covariance matrix adaptation evolution strategy (CMA-ES) has the best overall performance.

## 1. Introduction

Brain-computer interface (BCI) is defined as an alternative communication pathway which translates the measured brain activity into control commands (Pfurtscheller et al., 2008). Among the existing brain activity measurement techniques, EEG has been extensively employed for BCI applications due to its non-invasiveness, ease of implementation, and cost-efficiency (Lotte et al., 2007). Recently, the dry-electrodes and wireless EEG systems have also been studied in various experimental settings which has further expanded the scope of EEG based BCI systems (Tangermann et al., 2012; Gao et al., 2014).

The motor imagery has been one of the successful methods for EEG based BCI systems (McFarland et al., 2010; Hill et al., 2014). The application of motor imagery task to BCI systems has enabled subjects to sufficiently control a moving cursor in 2D space or a quadcopter in 3D space (Wolpaw et al., 2004; LaFleur et al., 2013). As shown in Muller-Gerking et al. (1999) and Guger et al. (2003), a contra-lateral amplitude decrease in α band can be found in most subjects during motor imagery tasks. Therefore, the motor imagery based BCI systems require transforming the recorded EEG signal into frequency domain in quasi real-time. Then the extracted frequency domain features are fed to machine learning algorithms for classification (Lotte et al., 2007; Robinson et al., 2013).

As frequency analysis of EEG is a major concern, many approaches such as the band pass filter bank, autoregressive (AR) model, Fourier transform, and Wavelet transform have been employed (Turnip and Hong, 2012; Wang et al., 2012b; Robinson et al., 2013; Hsu, 2014; Khan et al., 2014). However, all these methods have certain limitations. The frequency resolution of a band-pass filter is constrained by the order of the filter. The AR model coefficients only provide limited frequency information as the envelope of the spectrum reconstructed from AR coefficients is limited by the order of AR model (Shumway and Stoffer, 2011). The discrete wavelet transform is considered as a band-pass filter bank with orthogonal basis that has better frequency and temporal resolution (Wang et al., 2013b). However, the obtained wavelet coefficients are redundant in general. Further, post-processing is required to stabilize the performance of the classifier (Qin and He, 2005).

The EEG signal considered for BCI is generally band-limited. To decompose such signal into frequency domain and to maintain the balance between temporal and frequency resolution, the band-limited multiple Fourier linear combiner (BMFLC) has been developed (Veluvolu et al., 2012). BMFLC models the signal by adopting a truncated Fourier series. Adaptive filter algorithm is then employed to obtain amplitude estimation of individual frequency components in a pre-fixed frequency band. For application to motor imagery, BMFLC is applied to provide time-frequency mapping of the μ rhythm. The estimated amplitude of each frequency is then fed to a classifier (Wang et al., 2012a, 2013b). It was found in Wang et al. (2012a) that the performance of the BMFLC based BCI systems is improved if a subject-specific narrow band can be identified within the range of μ rhythm. The identification criterion and existence of such band have been established in Veluvolu et al. (2012). The reduced dimensionality in the feature vector resulted in better performance of the classifier.

Earlier approaches based on BMFLC only employed EEG signals that were recorded from sensorimotor cortex (i.e., C3 and C4 from international 10/20 system, Jurcak et al., 2007; Wang et al., 2012a, 2013b). Since only two EEG channels were considered, it was straightforward to perform the subject-specific band identification on the averaged time-frequency mapping (Wang et al., 2013b). Although the two channel system is simple in implementation, there is very limited scope for performance improvement. Due to the volume conduction of skull and scalp, the signal generated by the cortex of interest may be distributed to a vicinity of the area on the scalp and picked up by surrounding electrodes with different amplitude (Blankertz et al., 2008). To further enhance the classification performance, more data from EEG channels is often required for more complex problems.

The increase in the number of EEG channels poses two challenges. First, as the number of channel increases, direct identification of the subject-dependent reactive band becomes infeasible and an automated identification process is required. Secondly, as the feature vector includes all frequency estimates from all the channels, the dimension increases drastically. High dimensionality in feature vector will effect the performance of the classifier (Muni et al., 2006).

The above mentioned two problems are related as they both require optimization of the features to reduce the overall dimensionality and preserve the class-related information. For BCI application, the common spatial filter (CSP) is usually employed to solve the above two problems by reducing the whole EEG montage to a few spatial filtered channels in which the task-related information is significant (Lotte, 2014; Meng et al., 2015). The CSP works to find a projection that maximizes signal variance in one class while minimizing the variance in another class (Lotte and Guan, 2011). It has been shown in Wang et al. (2013a) that the application of the Tikhonov regularized CSP (TRCSP) to pre-process the EEG signal, and followed by the application of BMFLC for feature extraction provides a better performance as compared to the common average referencing or variant of Laplacian based spatial filters. As BMFLC decomposes the scalar measurement of each EEG channel into a multi-dimensional frequency estimation, the spatial filter that is optimized with respect to the bandpass filtered EEG signal may not be optimal for all frequency components.

In general, the above mentioned problems can be considered as a feature optimization problem (Chen et al., 2009; Estévez et al., 2009). However, the popular greedy search-based feature selection algorithm are not ideal to deal with multi-EEG channels due to its computational complexity. In the worst case scenario, to identify a optimal subset of a *n*-dimension feature vector, it would require all possible combinations of the *n*-dimension vector and that results in repetition of the cost function evaluation by $\overset{n}{\underset{r=1}{\sum }}C_{r}^{n}$ times. With increase in *n*, it will be no longer feasible due to computational load. As an alternative, the EA-based feature optimization, which encodes a feature subset in its solution and then optimizes it with respect to the performance index of a classifier, could be employed (Muni et al., 2006; Aberg and Wessberg, 2007; Aler et al., 2012). The computation load of the EA-based algorithm is limited by the number of maximum generations or function evaluations. Further, it is less intensive in computation demand as compared to the greedy-search based algorithms. A genetic type EA-based feature optimization algorithm is popular for the binarized solution to find the optimal feature subset (Banerjee et al., 2007). Due to this reason, it is not suitable for optimizing real-valued function. With the recent development of real-valued EA, the classifier optimization and the feature selection can be performed in one setting (Aberg and Wessberg, 2007).

The application of EA also provides us with a new way of estimating the spatial filter. As the common spatial filter estimation is a maximization process, the cost function is generally limited to a convex form for efficient application of the existing optimization algorithms (Lotte and Guan, 2011). With the help of real-valued EA algorithms, direct optimization of the spatial filter with respect to the classification accuracy can be performed where the convexity may not be preserved in the cost function. In Aler et al. (2012), the spatial filter was estimated by using the covariance matrix adaptation evolution strategy (CMA-ES), a type of EA.

In this work, we employ two real-valued EAs for optimizing the features that are produced by BMFLC in a multi-channel EEG. Both spatial filter and feature selection are encoded in the solution vector and optimized simultaneously. Two real-valued EA's, namely global and local real-coded genetic algorithm (GLGA) and CMA-ES, are employed for optimization. The above two algorithms are selected due to their stable performance on various real-valued practical problems (García-Martínez et al., 2008; Hansen et al., 2009). The result of the proposed EA-based feature optimization (BMFLC-EA) is compared with the BMFLC with CSP, and BMFLC. Our results show that the BMFLC with EA outperforms the rest. The preliminary results of this work has been published in Wang et al. (2015). In the current version, we have modified the optimal number of spatial filters selection procedure, and provide a comprehensive comparison with the existing methods.

The remaining paper is organized as follows. Three configurations of BMFLC based classifiers are proposed in Section 2, where the necessary details of the algorithm are provided. The performance of the proposed method and comparison to the existing methods are presented in Section 3. Section 4 concludes this paper.

## 2. Methods

In this section, three different configurations are proposed that are based on BMFLC for feature extraction. Various methods employed for this purpose are described in brief.

### 2.1. Proposed BCI architecture

**Figure 1:**
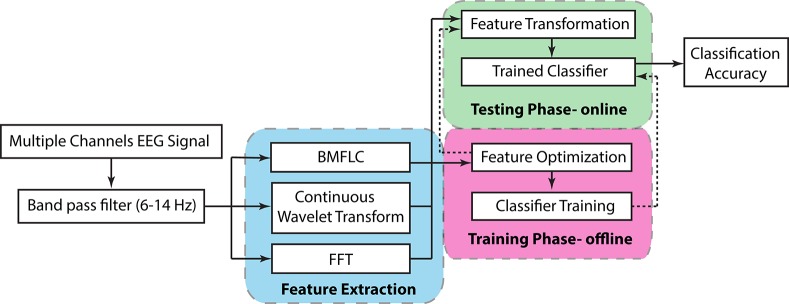
**Overview of the EEG signal processing chain**.

An overview of the EEG signal processing employed in this work is shown in Figure 1. The BCI system can be divided into a training phase and a testing phase. The feature optimization and classifier training is executed during the training phase. Once the optimal feature set and classifier has been obtained, the system can shift to the testing phase where feature optimization is no longer required. The reported classification accuracy is estimated during the testing phase of the BCI system. Before application to any feature extraction and optimization algorithm, the multiple channel EEG signals are first band-pass filtered with a Butterworth fifth order filter to the desired frequency band. To analyze the motor imagery, the μ rhythm is employed in the current study.

Three configurations that are based on BMFLC are discussed in this work. The major differences between three BCI configurations lie in how the spatial filter is employed in combination with the BMFLC. The BMFLC is employed to obtain time-frequency mapping of the EEG signal in each configuration. The spatial filter is employed to reduce the number of electrodes to a smaller subset. It can substantially reduce the dimension in the feature vector.

**Figure 2:**
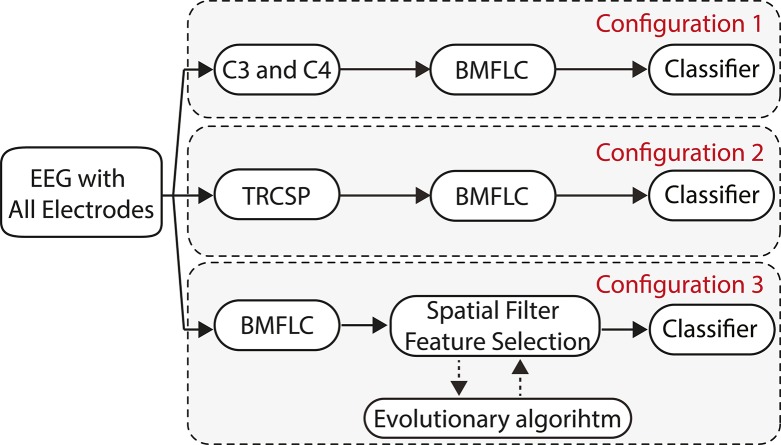
**Proposed configurations**.

The work flow of the three configurations is shown in Figure 2. Configuration 1 shown in Figure 2 is the simplest of the three configurations. As it is well-known that channels C3 and C4 in 10/20 systems have distinct patterns during motor imagery tasks, EEG signal from these two channels are selected. Thus, the spatial filter is formulated by a manual selection of channels in this configuration. The BMFLC is directly applied to the EEG data of C3 and C4.

Second configuration uses the Tikhonov regularized common spatial filter (TRCSP) to reduce the number of electrodes. Prior to the application of TRCSP, the EEG signal of each channel is band-pass filtered into the frequency band of interest, i.e., μ band. TRCSP then uses the band-pass filtered EEG to estimate spatial filters by maximizing the signal variance among classes. The number of spatial filters that are finally employed in the testing phase depends on the end application. The regularization parameter “α” in TRCSP is optimized based on the training data using the procedure developed in Lotte and Guan (2011). Later, the raw EEG signal in the testing set is multiplied by the selected spatial filters. The BMFLC is then employed to decompose the spatial filtered EEG signal to form the feature vector for the classifier.

Configuration 3 incorporates EA for estimating the spatial filter. In comparison with the earlier two configurations, the BMFLC is applied ahead of the spatial filter estimation. The dimension of the decomposed signal from BMFLC contains the full spectrum information with high dimensionality. EA is thus employed to find spatial filter in order to reduce the dimensionality of the decomposed signal. Compared to the optimization of the spatial filter in configuration 2, the optimization criterion in EA is the classification performance rather than the variance of the EEG signal in different classes. The importance of this difference will be more clear in the Results Section. Moreover, to further reduce the dimensionality of the feature vector, feature selection is also conducted by finding a subset of frequency components in the whole band that is provided by the BMFLC with EA.

### 2.2. BMFLC-KF based EEG feature extraction

Common to all three configurations, the BMFLC is applied to decompose the EEG signal to obtain time-frequency mapping of the α band. The amplitude information of each frequency component at each time instant or averaged over a fixed moving window can be employed to form the feature vector for the classifier.

BMFLC divides a pre-defined frequency band [ω_1_, ⋯ , ω_*n*_] into *n* equally distributed divisions with frequency spacing Δ_*f*_, and estimates the amplitude of each frequency component by using the Kalman filter as shown below (Wang et al., 2012b):

(1) $$y_{k}=x_{k}^{T}w_{k}+v_{k}$$

(2) $$w_{k+1}=w_{k}+\eta_{k}$$

where **x**_*k*_ and **w**_*k*_ are defined as

(3) $$x_{k}=\left\{\begin{array}{l} {\left[\begin{array}{cccc} \sin(\omega_{1}k) & \sin(\omega_{2}k) & \cdots & \sin(\omega_{n}k) \end{array}\right]}^{T}\\ {\left[\begin{array}{cccc} \cos(\omega_{1}k) & \cos(\omega_{2}k) & \cdots & \cos(\omega_{n}k) \end{array}\right]}^{T} \end{array}\right\}$$

(4) $$w_{k}=\left\{\begin{array}{l} {\left[\begin{array}{cccc} a_{1k} & a_{2k} & \cdots & a_{nk} \end{array}\right]}^{T}\\ {\left[\begin{array}{cccc} b_{1k} & b_{2k} & \cdots & b_{nk} \end{array}\right]}^{T} \end{array}\right\}$$

Assume that *v*_*k*_ and η_*k*_ are independent Gaussian processes with 0 mean and covariances of *R* and **Q**, respectively. The Kalman filter is employed for the adaptation of weights estimate ${\hat{\text{w}}}_{k}=\text{E}\left[{\text{w}}_{k}|{\text{y}}_{k-1}\right]$. The detailed implementation of BMFLC-KF is provided in the Supplementary Material (S.2). The weight vectors of BMFLC represents the Fourier coefficients of the band-limited signal. The estimated weights are further combined as follows:

(5) $$W_{k}={\left[\begin{array}{ccc} \sqrt{a_{1k}^{2}+b_{1k}^{2}} & \cdots & \sqrt{a_{nk}^{2}+b_{nk}^{2}} \end{array}\right]}^{T}$$

where **W**_*k*_ is the absolute weight vector of the frequency components at time instant *k*. The time-frequency mapping of a given signal is obtained by cascading the weights in **D** = [**W**_1_, …, **W**_*n*_].

### 2.3. EA-based spatial filter and feature selection optimization

As the usage and implementation of TRCSP in configuration 2 is straightforward, for the sake of brevity, the details are provided in Supplementary Material (S.1). In this subsection, we focus on developing EA-based spatial filter and feature selection optimization.

As EA has no limitations in the formation of solution vector, we need to encode the spatial filter and feature selection into the solution vector and then employ EA to optimize the solution vector with respect to the same cost function. Since the final objective of applying both spatial filter and feature selection is to improve the performance of the classifier, the cost function is selected to be the error rate of the classifier.

In this work, the covariance matrix adaptation evolution strategy (CMA-ES) and, global and local real-coded genetic algorithm (GLGA) are used due their superior performance in real-valued optimization (García-Martínez et al., 2008; Hansen et al., 2009). The algorithms for GLGA and CMA-ES are discussed in the following subsection. For more detailed information, please refer to S.3 and S.4 in Supplementary Material, respectively.

#### 2.3.1. Global and local real-coded genetic algorithm (GLGA)

Although we can encode a real-valued quantity into a binary string via some transformation and using traditional genetic algorithm (GA) (Hansen et al., 2009), the dimension of the solution becomes large as the number of required spatial filter grows. In this work, the GLGA, a real-valued version of GA, is employed (García-Martínez et al., 2008).

GLGA uses several operators to balance the exploration and exploitation. First, the solution vector in the current population is divided into male and female group by using female and male differentiation (FMD) operator. GLGA then uses parent-centric BLX-α (PBX-α) crossover operator to generate offspring. The PBX-α takes one solution vector from each group, the generated offspring that lies in the vicinity of the female solution. The distance between the offspring and the female solution is governed by the selected male solution and a parameter α. The parameter α determines the spread of offspring to its parents.

The uniform fertility selection (UFS) and the negative associative mating (NAS) are employed for the selection of female and male parents, respectively(García-Martínez et al., 2008). To apply UFS, a number of elite solutions in the current population are selected as a female parent. The number of times that a solution vector is used as female is tracked during the evolution. The less frequent solution is then selected as female parent. For selection of the male parent, the roulette wheel methods are applied to selected five candidate male parents. The Euclidean distance between the selected female parent and each of the possible male parent is calculated. The one with the highest distance is then selected as the male parent.

GLGA balances the global and local search by tuning the number of female and male parents. Let *N*_*m*_ and *N*_*f*_ denote the number of male and female parents, respectively. With a large *N*_*f*_, GLGA focuses on exploring the solution space, whereas a large *N*_*m*_ will restrict GLGA to concentrate on local area. GLGA is not operated by generation, rather it uses function evaluation as the criterion to shift from global to local search. GLGA-n% indicates that n% of function evaluations are used for global search and rest are used for local search. After certain number of function evaluations, GLGA assumes that the solutions have already entered into the vicinity of the global optimal, it thus reduces *N*_*f*_ to fine tune the solution in that region. To maintain the population size unchanged during evolution, the “replace the worst strategy” is adopted. Each time when an offspring is generated, the fitness value of the offspring is compared to the solution in the current population. If it is better than any of the solution in the current population, the worst one is replaced.

#### 2.3.2. CMA-ES

CMA-ES uses the second order statistics estimated from the solution space to find the optimal value of the target function. It is thus well-suited for optimizing the function with a complicated structure. It has been applied to both artificial functions and real practical problems and has shown to be performing better than the contemporary counterparts (Hansen et al., 2009; Elteto et al., 2012).

The (μ, λ) CMA-ES requires λ number of solutions in each generation, and select the best μ number of solutions for updating the value in next step. The CMA-ES algorithm generates the solution $s_{k}^{\left(g\right)}$ by sampling from a Gaussian distribution with mean *m*, step-size δ and covariance estimation **C**, where *g* and *k* are indexes for generation and solution, respectively. To find the solution for the next generation, CMA-ES estimates the mean, step-size, and covariance with the solution in the current generation considering the history path of the solution evolution. After each generation, the solutions are ranked based on their fitness value. The update of mean is carried out by a weighted summation of the μ best solutions in the current generation. The value of **C** is determined by the covariance of the estimated evolution path denoted by *p*_*c*_ and the covariance estimated from the μ number of solutions in the current generation.

#### 2.3.3. Optimization of spatial filter and feature selection

The key to the application of EA for optimizing the spatial filter and feature selection simultaneously is to develop a proper solution vector. The spatial filter is real-valued by default, unlike the genetic algorithm based feature solution. The binary valued feature selection is replaced with real-valued problem. It was shown in earlier studies that the subject-specific band has a maximum bandwidth of 3 Hz (Veluvolu et al., 2012). Therefore, the EA is employed to search for the starting frequency and the optimal bandwidth of the subject-specific band. Moreover, the dimensionality of the solution vector is significantly reduced by encoding the frequency selection in real-value compared to the binary encoding scheme. As the number of function evaluations required for a EA to converge depends on the dimension of the solution vector (Herrera et al., 1998), this approach should be able to reduce the computational complexity and further improve the solution quality when a fixed number of function evaluations is considered.

Thus, the solution vector for GLGA and CMA-ES is constructed as follows:

$$sol=[\overset{\text{Spatial Filter}}{\overset{︷}{sf_{11},\ldots ,sf_{1M},\;sf_{21},\ldots ,sf_{2M},\ldots }},\overset{\text{Frequency Selection}}{\overset{︷}{FS,\;BW}}]$$

where *sf*_*ij*_ denotes the *j*th component in *i*th spatial filter, *FS* is the starting frequency, and *BW* is the bandwidth. *M* is the total number of EEG channels. The number of resultant spatial filters depend on the application. For motor imagery BCI system, the number of classes is usually chosen as the number for spatial filters selection (Aler et al., 2012). As the EA is applied to the time-frequency mapping of BMFLC, *FS* has to lie in the range of [*f*_1_, *f*_*n*_ − *BW*], where *f*_1_ and *f*_*n*_ are the lower and upper cutoff frequency of BMFLC, respectively. As shown in Veluvolu et al. (2012), *BW* is constrained to have a maximum 3 Hz band.

The classification error obtained from linear discriminant analysis (LDA) is employed as the optimization criterion for GLGA and CMA-ES. The frequency weights that are estimated from BMFLC-KF are used as the original feature set. To optimize the spatial filter and frequency selection, the frequency weights obtained from each channel is first combined to the following form:

(6) $$F_{k}={\left[\begin{array}{ccc} w_{k,1}^{fs} & \ldots & w_{k,M}^{fs}\\ \vdots & \vdots & \vdots \\ w_{k,1}^{fe} & \ldots & w_{k,M}^{fe} \end{array}\right]}^{T}$$

where $w_{k,M}^{fs}$ indicates the weights of frequency *fs* at time instant *k* in channel *M*. Then, the best solution vector in the current generation is separated into two parts, frequency selection and spatial filter. The optimal frequency band is extracted form the original feature space by setting *fs* = *FS* and *fe* = *FS*+*BW*. The spatial filter component of the solution vector is re-organized as follows:

(7) $$SF={\left[\begin{array}{ccc} sf_{11} & \ldots & sf_{1M}\\ sf_{21} & \ldots & sf_{2M} \end{array}\right]}^{T}$$

To obtain the optimized feature set, the spatial filter **SF**^*T*^ is multiplied with the **F**_*k*_, where superscript *T* denotes matrix transpose. The resultant feature vector as well as the corresponding label are then sent to the classifier. The error rate of the employed classifier is used to guide the EA to search for a better spatial filter and feature set. To avoid over-fitting, only the classification error obtained from the validation data is used. The solution vector obtained at the end of the evolution contains the optimized spatial filter and frequency selection.

### 2.4. Dataset description

To test the performance of the proposed architecture, we choose two publically available BCI datasets. The first dataset (denoted as Dataset I) is from Brain Computer Interface Competition IV (Brunner et al., 2007). Dataset I contains EEG data of nine subjects. EEG was recorded from 22 Ag/AgCl electrodes sampled at 250 Hz. All signals were recorded monopolarly with the left mastoid as reference and right mastoid as ground. Four classes of cue-based motor imagery tasks were carried out, namely the imagination of the movement of the left hand, right hand, both feet and tongue. Each subject data was recorded in two sessions on separate days. Each session consisted of six runs separated by short breaks. One run consisted of 48 trials. During the recording, the subjects sat on a comfortable armchair in front of a computer screen. At the beginning of each trial (*t* = 0*s*), a fixation cross appeared on the black screen. Two seconds later, a cue in the form of an arrow pointing either to the left, right, down, or up displayed on the screen and lasted 1.25*s*. The subjects were asked to perform the motor imagery task until the fixation cross disappeared from the screen at *t* = 6 s. For more details about data collection, see Brunner et al. (2007).

The second dataset (denoted as Dataset II) was taken from the BCI competition 2003, dataset 2a (McFarland et al., 1997). The dataset contains motor imagery data from three subjects. All subjects were informed to modulate their sensorimotor rhythm to move a cursor from the left edge of the screen to one of the four designated locations appeared on the right edge of the screen. In this paper, we chose the top and bottom target out of totally four available targets. Totally, 64 channels EEG signal were recorded according to the international 10/20 system with a sampling frequency of 160 Hz. Each subjects participated 10 sessions of the experiment. In this work, the top and bottom target in the training session (session 1–6) was employed.

## 3. Results

### 3.1. Experiment settings

As the EEG α band is of interest, the frequency range for BMFLC-KF is set to *f*_1_ = 6 Hz and *f*_*n*_ = 14 Hz. Δ*f* is set to 0.5 Hz as it has been shown to offer better results in EEG signal decomposition (Wang et al., 2012b). For applying CMA-ES algorithm, the population size λ is set to be five times the solution dimension. The number of spatial filters are selected to be equal to the number of classes in the dataset. The reason for this selection will be discussed in the later section.

The CMA-ES starts by setting the initial value to *s*^(0)^, δ^(0)^, **C**^(0)^, and updating the solution and is similar to the implementation provided in Hansen and Kern (2004). The parameters provided in Hansen et al. (2009) are employed as they are shown to offer robust performance.

For GLGA, the parameters are chosen from García-Martínez et al. (2008). α is equal to 0.5 and 25% of function evaluations are employed for global search. For a fair comparison between CMA-ES and GLGA, the functional evaluation of GLGA is set to 10,000, as it approximately matches to the function evaluations used by CMA-ES. *N*_*m*_ = *N* and *N*_*f*_ = *N*/2 are used for global searching and *N*_*m*_ = 100 and *N*_*f*_ = 5 are employed for local searching, where *N* is the population size. The algorithm terminates when the stopping criterion is met.

For performance analysis, the EEG dataset is divided into training set and testing set according to 10-times cross validation scheme. Feature optimization including CSP and EA based methods are performed on the training set. The same training set is employed to train the final classifier. The classification accuracy which is obtained on the testing data is employed for performance comparison. It is important to note that, since the GLGA and CMA-ES relies on classification error as the cost function, a separate 10-times cross validation is also applied to the training set.

Another important factor that affects the performance is the number of spatial filter pairs employed for each algorithm/configuration. In this paper, the number of spatial filter pairs is selected for each algorithm separately to ensure optimal performance for each configuration. The rationale and procedure for this selection will be discussed in the later section.

For the sake of comparison, we consider other feature extraction and optimization algorithms that have been reported to have superior performance on BCI motor imagery tasks. The continuous wavelet transform (CWT) with was shown to have accurate time-frequency resolution in analyzing the EEG signal (Hsu, 2015). Thus, it has been included in our comparison analysis. We have also chosen Daubechies wavelets of order 4 (denoted as Db4) as suggested in Hsu (2015). The obtained wavelet features undergoes the same feature optimization procedure with CMA-ES (denoted as Db4-CMAES). Further, the filter bank common spatial pattern (FBCSP) is included as it was shown to have superior performance on the BCI competition dataset (Ang et al., 2012). In this work, we have implemented a BMFLC based FBCSP. In summary, the EEG signal is first decomposed by the BMFLC. Then, CSP filter was employed for each frequency estimate and the features were constructed as the logarithm power. The obtained features were then optimized by employing the mutual information criterion (Ang et al., 2012). Further, the algorithm developed in Aler et al. (2012) is also implemented (denoted as FFT-CMAES).

### 3.2. Performance of GLGA and CMA-ES based feature optimization

**Figure 3:**
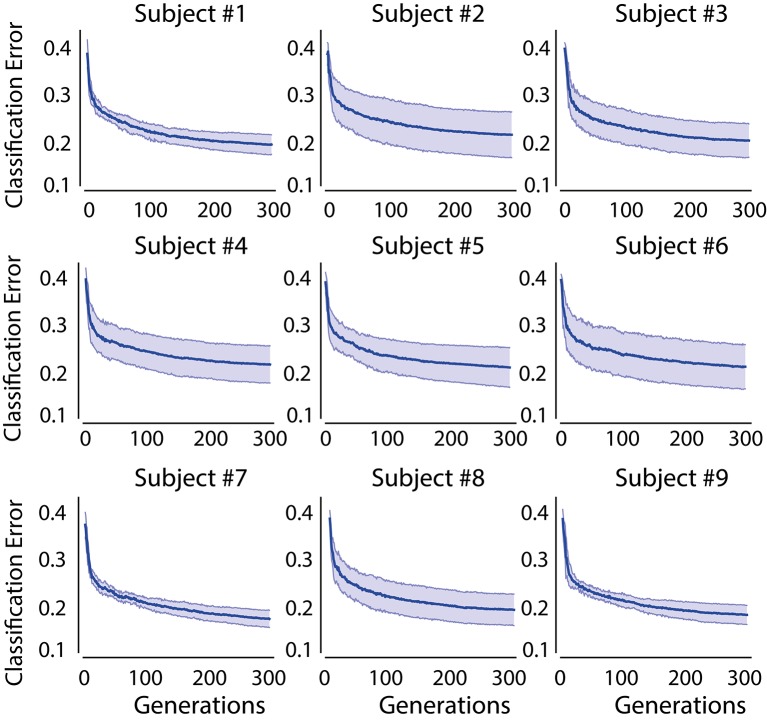
**Evolution of training of CMA-ES for 300 generations**. Shaded area indicates the standard deviation obtained from 10 cross validation runs.

The value of the cost function of CMA-ES for each generation is shown in Figure 3 for all subjects. The shaded area indicates the standard deviation of the classification error obtained from 10-times cross validation scheme. For all the subjects, we observe that the classification error converges to a stable bound at the end of the evolution process. It can be noted that with CMA-ES, the error converges at a faster rate in the first 20 generations. Later on, the convergence rate is slowed up to 100 generations. After 100 generations, the classification error becomes stable. The simulations for 500 generations also yielded similar results.

Another observation from Figure 3 is that the standard deviation varies significantly across subjects. A small standard deviations can be identified in subject-1, -7, and -9. Whereas, large standard deviation can be observed in the rest of the subjects. The large standard deviation implies that CMA-ES converges to different values at cross validation runs. Further, it may be also due to the complexity of the cost function employed. However, this divergence in the evolution process does not affect the performance of the final classifier with CMA-ES as shown in **Figure 7**. This result indicates the superiority of CMA-ES in the optimization of a complex function as compared to GLGA.

**Figure 4:**
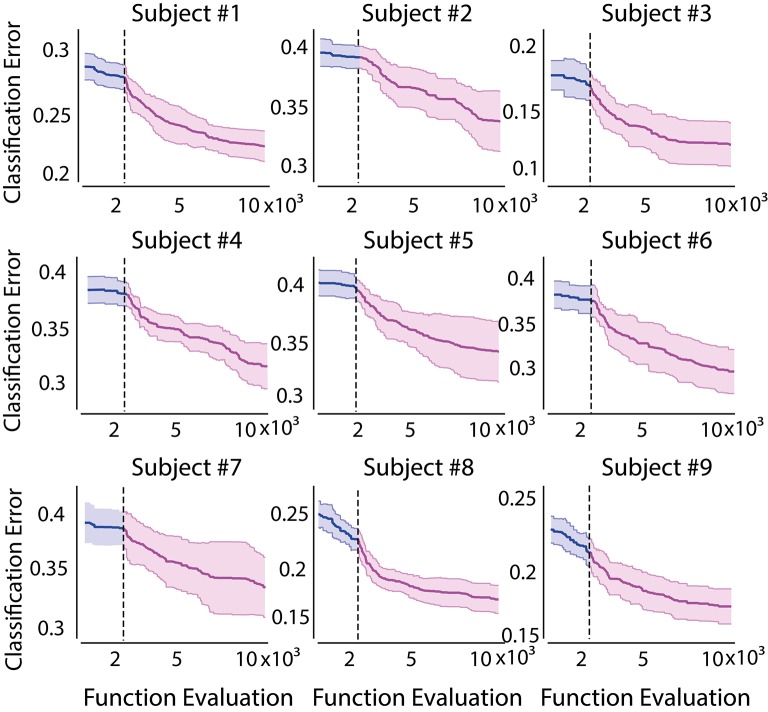
**Evolution of training of GLGA-25 for 10,000 function evaluations**. Shaded area indicates the standard deviation obtained from 10 cross validation runs. The vertical line indicates the transition from global searching to local searching.

Similarly, the cost function evolution of GLGA is shown in Figure 4. The vertical line in Figure 4 indicates the transition from global search to local search. The first observation is the slow error decay during the global search phase. After GLGA moves to local search, initially, a fast error decay followed by a stable phase can be observed. The standard deviation is generally small during global search and the value increases when the algorithm approaches the end of the execution. The results suggest that GLGA tends to converge to different area after each global search during each cross validation run.

Comparing the error evolution of GLGA to CMA-ES, we first note that the GLGA has a higher standard deviation over all subjects, whereas the error evolution in CMA-ES varies with the subject with a smaller volatility. Moreover, CMA-ES converges to a small error bound at the end of the evolution process as compared to GLGA.

**Figure 5:**
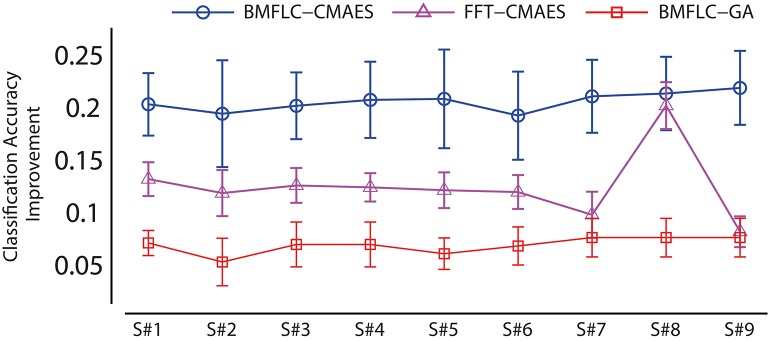
**Classification accuracy improvement *BMFLC-CMAES, FFT-CMAES, and BMFLC-GA***.

To further evaluate the performances of GLGA and CMA-ES, the overall error improvement of each algorithm is obtained for all subjects. The error improvement is calculated as the difference between the maximum error and the minimum error obtained during evolutionary process. It shows how well the employed EA explores the error surface. For comparison, the algorithm proposed in Aler et al. (2012) (named as FFT-CMAES) is also implemented and the results are shown in Figure 5. We observe CMA-ES based feature optimization outperforms the GLGA in terms of performance improvement. The BMFLC-CMAES shows superiority in performance compared to FFT-CMAES. BMFLC-GA has the lowest improvement during training. The error improvement is stable for all the subjects with FFT-CMAES, as shown by small standard deviation.

### 3.3. Performance of the final classifier for all configurations

**Figure 6:**
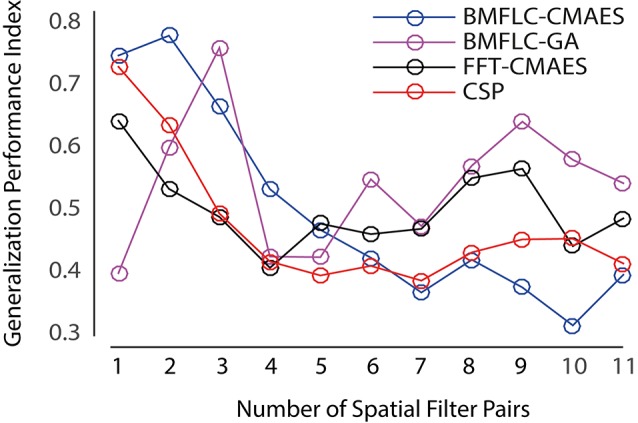
**Selection of optimal number of spatial filter pair**.

To perform the comparison for all different configurations, the number of spatial filters required for each configuration needs to be identified. As the dataset employed in this work consists of 22 EEG recordings for motor imagery tasks, we can therefore obtain up to 11 pairs of spatial filters for each subject. In this work, we select the number of spatial filters for each configuration based on the generalization performance index. This index is used to quantify the performance for the selected number of spatial filters over all subjects. To calculate this index for a configuration, we first obtain the classification accuracy for all subjects and all spatial filter pairs in the training data set. Then for a particular subject, the classification accuracy for all spatial filter pairs is normalized to the range of [0 1]. The employed unity-based normalization ensures that the optimal spatial filter pair for each subject has the performance index of 1, so that the difference in classification accuracy among subjects will not bias the spatial filter selection. Then, the generalization performance index for each spatial filter pair is the normalized classification accuracy averaged over all subjects. The results of generation performance index for all configurations is shown in Figure 6.

From Figure 6, we can observe that all configurations tends to have better generalization performance for fewer number of spatial filter pairs. The CSP and FFT-CMAES display maximum generalization performance when one pair of spatial filters is employed. BMFLC-CMAES peaks at two pair spatial filters, whereas BMFLC-GA requires three pairs of spatial filters. Thus, the obtained number of spatial filter pair is set to each configuration for comparison. After optimizing the features by employing CSP, CMA-ES, and GLGA with pre-selected parameters and number of spatial filter pairs, a final classifier is then built with the same training set and then finally evaluated on the testing set.

**Figure 7:**
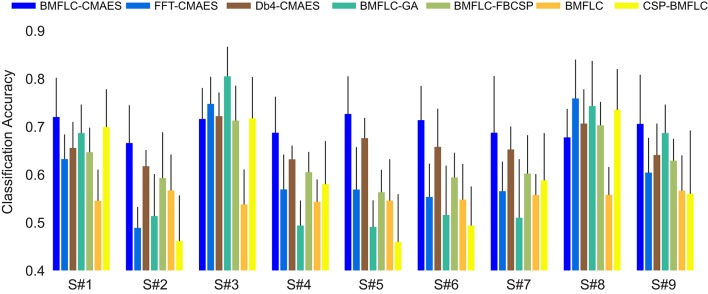
**Classification accuracy on testing set of all configurations on Dataset I**.

The performance of all subjects in Dataset I averaged over 10-times cross validation is shown in Figure 7. Configuration 1 is denoted as BMFLC where no feature optimization has been employed. Configuration 2, where CSP is applied prior to BMFLC is denoted by CSP-BMFLC. The two variants of EA based feature optimization procedures are named as BMFLC-CMAES and BMFLC-GLGA, respectively.

In Figure 7, we observe that BMFLC-CMAES outperforms all other algorithms in seven out of nine subjects. Further, it has also the best performance when averaged over all subjects, and passes the Friedman test with α = 0.05. We also observe that the features obtained from BMFLC and later optimized by CMA-ES and GLGA display better performance compared to FFT-CMAES on average. The BMFLC-FBCSP has better performance as compared to BMFLC and CSP-BMFLC. The performance varies across subject in CSP-BMFLC and is subject-dependent. It is also clear that feature optimization improves the overall performance.

The classification performance with Dataset II is shown in Table 1. We observe that the BMFLC-CMAES and Db4-CMAES outperformed all other methods in mean accuracy. This observation clearly demonstrates the superiority of employing time-frequency decomposition for feature extraction. Further, we notice that the BMFLC-GA is inferior to the BMFLC-FBCSP method.

**Table 1:** **Classification accuracy on testing set of all configurations—Dataset II**.

**Subject No**.	**Classification accuracy**						
	**BMFLC-CMAES**	**FFT-CMAES**	**Db4-CMAES**	**BMFLC-GA**	**BMFLC-FBCSP**	**BMFLC**	**CSP-BMFLC**
Subject AA	0.83 ± 0.03	0.73 ± 0.08	0.80 ± 0.07	0.75 ± 0.12	0.76 ± 0.13	0.58 ± 0.15	0.69 ± 0.08
Subject BB	0.81 ± 0.04	0.78 ± 0.08	0.80 ± 0.07	0.67 ± 0.10	0.76 ± 0.11	0.68 ± 0.13	0.74 ± 0.09
Subject CC	0.83 ± 0.05	0.75 ± 0.07	0.78 ± 0.06	0.75 ± 0.07	0.74 ± 0.08	0.68 ± 0.11	0.71 ± 0.16
Average	0.83 ± 0.04	0.76 ± 0.08	0.80 ± 0.08	0.72 ± 0.10	0.76 ± 0.10	0.65 ± 0.13	0.72 ± 0.11

**Figure 8:**
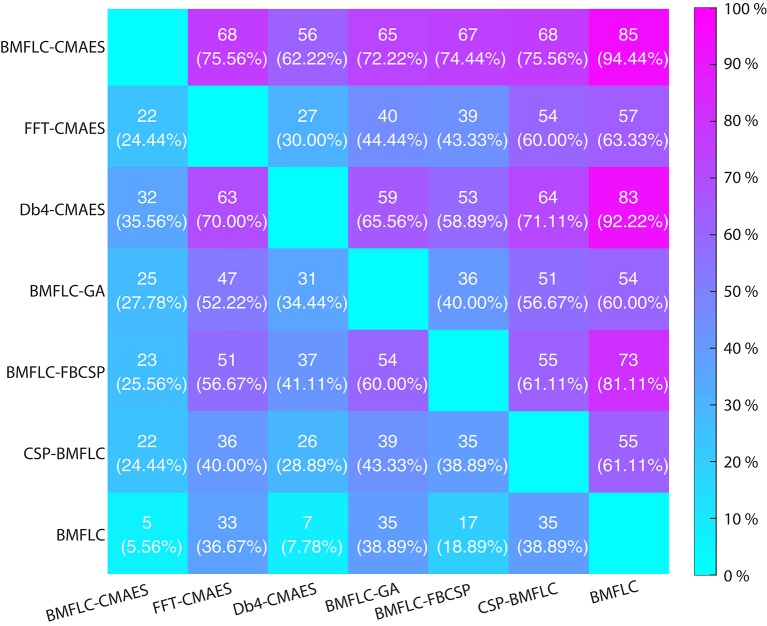
**Performance comparison for various configurations on Dateset I**.

We also compared the number of occasions of one algorithm outperforming another algorithm by accumulating the results from the cross validation runs of all subjects in Dataset I. Since 10 cross validation were employed, a total of 90 (9 subject × 10 cross validations runs) accuracy estimates per configuration were obtained. The results are shown in Figure 8. The total number and its corresponding percentage value by which one algorithm outperforms the other algorithm is provided in the middle of each block.

From Figure 8, it clearly demonstrates the necessity of feature optimization. The configurations that are combined with feature optimization have better classification accuracy when BMFLC based features are employed. The results in the first and second row when compared to the third row of Figure 8 further demonstrate the superiority of BMFLC and Db4 in extracting information from EEG as compared to FFT. Moreover, we also observe that all configurations that involve feature optimization outperform the traditional methods. The high standard deviation obtained with GLGA (Figure 4) is also reflected in the classification results as its overall performance is lower compared to BMFLC-CMAES.

To further consolidate our observed results, we have performed a repeated measure of ANOVA by collecting all accuracy estimates from all methods on all subjects. As the obtained accuracy estimates violated the sphericity assumption, Greenhouse-Geisser correction has been employed. Results show that the mean accuracy differences are statistically significant among all methods [*F*_(4.037, 359.252)_ = 27.904, *p* < 0.01]. *Post-hoc* tests using the Bonferroni correction revealed that the proposed BMFLC-CMAES outperforms all comparison methods with *p* < 0.01. All methods with feature optimization show superior performance as compared to BMFLC (*p* < 0.05). It highlights the necessity of employing feature optimization in BCI applications. Furthermore, the BMFLC-CMAES and Db4-CMAES show statistically significant accuracy improvement over CSP-BMFLC and FFT-CMAES (*p* < 0.01), which indicates the merit of using time-frequency decomposition to expand the feature space.

### 3.4. Runtime complexity

To show the applicability of the proposed methods to the real-time BCI applications, we conducted a runtime computational complexity analysis. The reported runtime estimates for each algorithm are obtained on a system with Intel(R) Core i5-6500 CPU and 8GB RAM. The time duration was measured using Matlab (Mathworks, USA) inbuilt function. Here, we reported the average time duration to finish one validation run. The obtained runtime estimates for all feature optimization methods are given in Table 2.

**Table 2:** **Runtime complexity of all configurations**.

**Methods**	**BMFLC-CMAES**	**FFT-CMAES**	**Db4-CMAES**	**BMFLC-GA**	**BMFLC-FBCSP**	**CSP-BMFLC**
Time (s)	471.84 ± 19.33	515.69 ± 32.08	494.05 ± 47.13	17151.27 ± 839.07	1.55 ± 0.81	0.01 ± 0.01

The runtime requirement of the proposed feature optimization procedure consists of the feature extraction, optimization, and classification. The feature extraction method BMFLC has been shown to have superior computational complexity as compared to STFT and CWT (Wang et al., 2013b). In this work, we estimated that the time required for BMFLC to decompose a 2 s EEG epoch is 0.0349s ± 0.0037. Also, the computational demand of LDA is negligible as only linear operation is involved. Therefore, the main contributor to the computational complexity of the proposed algorithm is the time taken by the employed optimization algorithm.

All EA based feature optimization algorithms require few minutes of run-time, as shown in Table 2, to attain optimized features. Compared to the CSP based methods, the EA based feature optimization has much higher computational demands. However, the feature optimization is usually conducted during the training phase of the BCI application. The feature optimization only needs to be finished in a reasonable time. We estimated that the BMFLC-CMAES requires 7 min to find the optimized feature subspace. Once the optimized features have been identified during training, the system only needs to perform feature extraction and classification in real-time.

## 4. Discussion

In this work, different BCI configurations which employ BMFLC based features for classification have been developed. As our results suggest, BMFLC-KF with manually selected channels has the lowest classification performance. However, without any optimization, the BMFLC feature offers the most stable performance. There are two possible reasons for this. First, the BMFLC-KF decomposes the EEG signal with high accuracy and this provides the classifier with a stable feature set. Secondly, the stability in the classification results may also indicate the lack of diversity and limited information available within the two channels. This result also highlights the necessity for employing the optimization for feature extraction.

It is evident that the performance of CSP-BMFLC is subject dependent. For three out of nine subjects in Dataset I, the CSP-BMFLC has good testing accuracy. However, for the rest subjects, the CSP-BMFLC performance is similar to or even worse than applying BMFLC alone. As CSP algorithm relies on the training data to find the spatial filter, the unsatisfactory performance in test data may be due to over-fitting of the training data.

Comparing the results from the configurations which involve feature optimization, the BMFLC-CMAES outperforms Db4-CMAES, BMFLC-GA, FFT-CMAES, and BMFLC-FBCSP. In fact, BMFLC-CMAES, and Db4-CMAES have the best performance among all configurations considered in this work. Our results indicate that the feature optimization is critical in improving the testing performance for a given feature set.

We also observe that the performance of each EA involved configuration differs. BMFLC based system is degraded when GLGA is employed for feature optimization. As GLGA divides the evolution into two distinct phases, global, and local searches, the overall performance of GLGA depends on the quality of the global search phase. If the global search fails in finding the promising area, then the subsequent local search may not improve the results and this may introduce severe over-fitting. However, if the global search successfully enters into the promising area, then the local search can fine-tune the solution and this results in good performance as shown in subject 3 and subject 8 in Figure 7.

Among all feature optimization methods that employ BMFLC, the BMFLC-CMAES has the best performance. Whereas, the BMFLC-GA provides inferior performance as compared to BMFLC-FBCSP. This result indicates that the careful selection of optimization algorithm is also critical to identify the suitable feature subset. Our results suggests that the CMAES is preferable for feature optimization.

BMFLC-CMAES, Db4-CMAES, and FFT-CMAES differ only in the feature extraction algorithm. The superior performance of BMFLC-CMAES and Db4-CMAES as compared to FFT-CMAES highlights the ability of time-frequency decomposition in extracting the information from motion induced EEG signal as compared to the traditional FFT. Moreover, the BMFLC-CMAES provides better performance as compared to Db4-CMAES. However, the improvement failed to attain statistical significant level (*p* < 0.01). The current results are in line with our previous work that BMFLC has similar performance in terms of time-frequency decomposition of EEG signal (Wang et al., 2013b). However, as shown in Wang et al. (2013b), the BMFLC has less computational complexity as compared to CWT. Thus, it is more suitable for the real-time BCI applications.

The superiority in classification performance obtained from EA based feature optimization comes with a price. All EA based configurations require much higher computational resources compared to the rest of the configurations employed in this work. The computational complexity of the EA based feature optimization grows with the increasing number of EEG channels and the frequency band of interest. However, the frequency band for a BCI system is usually limited and the feature optimization is only required during training phase. We show that the required training time for BMFLC-CMAES is around 7 min, which is tolerable in the current BCI applications. Thus, the computational burden posed by the EA has little impact to the real-time applicability of the proposed feature optimization algorithm.

Feature optimization is mainly intended to identify a suitable subset of features from the original feature space which can improve the classification accuracy and mitigate the requirement in computational demand. The most reliable method to solve the feature optimization problem is to enumerate all possible feature combinations from the the original feature space. However, such enumeration is only possible when the number of features in the original feature space is small. It has been shown in a fNIRS based BCI application that by selecting two out of six features, the testing accuracy is maximized (Naseer et al., 2016). However, the feature dimension in the current work is 17 per channel. With a 22 channels EEG recording, it results in a 374 dimensional feature vector. To enumerate on all combinations on such high feature space requires high computational requirements. The case is even worse when the number of available channels increases. Therefore, an evolution algorithm based approach was desirable to optimize the features in such high dimensional space.

Classification performance of the BCI applications can be improved with support vector machine (SVM) as the classifier (Schlögl et al., 2005; Naseer, 2014; Khan and Hong, 2015). The training of SVM is however computationally extensive when compared to a LDA classifier (Khan and Hong, 2015). Furthermore, to obtain better performance with SVM, its hyper-parameters need to be fine-tuned (Vapnik, 1995; Suykens et al., 2002), which increases the computational cost for training a BCI system considerably. The main aim of the current paper is to show the impact of the feature exaction and feature optimization on the performance of BCI system. Therefore, for the illustration of classification performance with proposed optimal feature selection technique, we chose LDA as the classifier in this work. For completeness, we report that one can chose any advanced machine learning techniques as a classifier for the proposed feature extraction and optimization scheme.

## 5. Conclusion

In this work, the performance of BMFLC-KF with various combinations of FBCSP, CSP, CMA-ES, and GLGA for feature optimization is quantified with a two-class BCI application. Two publically available BCI competition datasets are employed to study the performance of the proposed feature extraction and feature optimization algorithm. The evolutionary algorithms (namely CMA-ES and GLGA) are employed to optimize the spatial filter and feature selection simultaneously. It is clear from our results that the performance of the final classifier can be improved by employing any of the above mentioned feature optimizing algorithms. Statistical analysis reveals that the proposed BMFLC-CMAES based feature optimization algorithm outperforms its counterparts. Moreover, the superior performance of time-frequency features obtained from BMFLC and CWT also highlights the necessity of employing time-frequency decomposition for feature extraction in BCI applications. The small execution time of CMA-ES indicates that it is more suitable for feature optimization as compared to GLGA.

## Author contributions

This work was carried out by YW under the supervision of KV.

## Funding

This research was supported by the Basic Science Research Program through the National Research Foundation of Korea (NRF) funded by the Ministry of Education, Science and Technology under the Grant NRF-2014R1A1A2A10056145 and in part by the BK21 Plus project funded by the Ministry of Education, Korea (21A20131600011).

### Conflict of interest statement

The authors declare that the research was conducted in the absence of any commercial or financial relationships that could be construed as a potential conflict of interest.
