# Supplementary material for: Evolutionary Algorithm Based Feature Optimization for Multi-Channel EEG Classification
Source: Front Neurosci. 2017 Feb 1;11:28. doi: 10.3389/fnins.2017.00028 (PMC5285364; doi:10.3389/fnins.2017.00028)
Supplement: Supplementary file 1 [file Presentation1.pdf]

# *Supplementary Material*: Evolutionary Algorithm based Feature Optimization for Multi-channel EEG Classification

**Yubo Wang and K. C. Veluvolu** *
*Correspondence:
K. C. Veluvolu
veluvolu@ee.knu.ac.kr

## 1 COMMON SPATIAL FILTER (CSP)

The common spatial filter (CSP) that proposed in (Samek et al., 2012; Lotte and Guan, 2011) is an optimization process that tries to find the coefficients $m$ that maximize the following cost function. In this work, the Tikhonov regularized CSP (TRCSP) is employed. The cost function of TRCSP is defined by:

$$J(w) = \frac{m^T C_1 m}{m^T C_2 m + \alpha m^T \mathbf{I} m} = \frac{m^T C_1 m}{m^T (C_2 + \alpha \mathbf{I}) m} \tag{S1}$$

where $C_i$ is the variance of EEG signal in $i$th condition, $\alpha m^T \mathbf{I} m$ is the penalty term and $\alpha$ is selected such that the performance of a LDA classifier is maximized with a 10-fold cross validation. The optimization of equation (S1) is done by solving the following Lagrange equation

$$L(\lambda, m) = m^T C_1 m - \lambda(m^T (C_2 + \alpha \mathbf{I}) m - 1) \tag{S2}$$

The solution of equation (S2) is equivalent to the eigenvalue problem that is given by $(C_2 + \alpha \mathbf{I})^{-1} C_1 m = \lambda m$. The spatial filter $m$ that maximizes the equation (S2) are the eigenvectors of $(C_2 + \alpha \mathbf{I})^{-1} C_1$.

## 2 BMFLC-KF

The BMFLC is a signal model that can be used to estimate the unknown amplitude in a pre-defined frequency band. The informative motor modulation of EEG signal, *i.e. ERD*, can be observed in $\alpha$ band. The previously developed BMFLC divides a pre-defined frequency band $[\omega_1, \cdots, \omega_n]$ into $n$ equally distributed divisions with frequency spacing $\Delta_f$, and estimates the amplitude of each frequency component by using least-mean-square algorithm(LMS) (Veluvolu et al., 2012; Wang et al., 2012a). Although the estimation accuracy is high, the LMS algorithm can not guarantee an accurate and fast tracking of amplitude variation. In order to improve the tracking performance of the existing BMFLC, we later developed Kalman filter based BMFLC (BMFLC-KF) (Wang et al., 2012b) was later developed.

The state-space equation of BMFLC is given as:

$$y_k = \mathbf{x}_k^T \mathbf{w}_k + v_k \tag{S3}$$

$$\mathbf{w}_{k+1} = \mathbf{w}_k + \eta_k \tag{S4}$$

where $\mathbf{x}_k$ and $\mathbf{w}_k$ are defined as:

$$\mathbf{x}_k = \begin{Bmatrix} \begin{bmatrix} \sin(\omega_1 k) & \sin(\omega_2 k) & \cdots & \sin(\omega_n k) \end{bmatrix}^T \\ \\ \begin{bmatrix} \cos(\omega_1 k) & \cos(\omega_2 k) & \cdots & \cos(\omega_n k) \end{bmatrix}^T \end{Bmatrix} \tag{S5}$$

$$\mathbf{w}_k = \begin{Bmatrix} \begin{bmatrix} a_{1k} & a_{2k} & \cdots & a_{nk} \end{bmatrix}^T \\ \\ \begin{bmatrix} b_{1k} & b_{2k} & \cdots & b_{nk} \end{bmatrix}^T \end{Bmatrix} \tag{S6}$$

With the assumptions that $v_k$ and $\eta_k$ are independent Gaussian process with 0 mean and covariance of $R$ and $\mathbf{Q}$ respectively. The Kalman filter iteration is given as

$$\mathbf{K}_k = \mathbf{P}_k \mathbf{x}_k^T [\mathbf{x}_k^T \mathbf{P}_k \mathbf{x}_k + R]^{-1} \tag{S7}$$

$$\hat{\mathbf{w}}_{k+1} = \hat{\mathbf{w}}_k + \mathbf{K}_k (y_k - \mathbf{x}_k^T \hat{\mathbf{w}}_k) \tag{S8}$$

$$\mathbf{P}_{k+1} = [\mathbf{I} - \mathbf{K}_k \mathbf{x}_k] \mathbf{P}_k + \mathbf{Q} \tag{S9}$$

where $\hat{\mathbf{w}}_k = \mathbf{E}[\mathbf{w}_k | \mathbf{y}_{k-1}]$ denotes the mathematical expectation of $\mathbf{w}$ at time instant $k$ with respect to previous observation $\mathbf{y}$ at $k-1$, $\mathbf{P}_k$ is the estimated state error covariance and $\mathbf{K}_k$ is the Kalman gain. The Kalman filter starts with the initial condition $\hat{\mathbf{w}}_0 = \mathbf{0}$ and $\mathbf{P}_0 = \mathbf{I}$. The weight vectors of BMFLC represents the Fourier coefficients of the band-limited signal.

## 3 GLOBAL AND LOCAL REAL-CODED GENETIC ALGORITHMS

As the function that needs to be optimized is unknown, the performance of GA depends on the ability of exploring the function space and then fine tuning the solution in a selected promising area. GLGA uses several operators to handle real-valued solution vector and also balances the global and local searching (García-Martínez et al., 2008).

For handling real-valued solution vector, the parent-centric BLX-$\alpha$ (PBX-$\alpha$) crossover operator is used. The PBX-$\alpha$ takes two real-valued solution vectors denoted as $\mathbf{X}_1 = (x_1, \ldots, x_n)$ and $\mathbf{Y}_1 = (y_1, \ldots, y_n)$, where $x_i, y_i \in [a_i, b_i] \subseteq \Re, i = 1, \ldots, n$. The offspring $\mathbf{Z} = (z_1, \ldots, z_n)$ is generated by the following rule:

$$z_i = \text{rand}(l_i, u_i) \quad \text{for} \quad i = 1, \ldots, n \tag{S10}$$

where $l_i = max(a_i, x_i - \mathbf{I} \cdot \alpha)$, $u_i = min(b_i, x_i + \mathbf{I} \cdot \alpha)$ and $\mathbf{I} = |x_i - y_i|$. The offspring $z_i$ is randomly picked up within the range of $l_i$ and $u_i$. The parameter $\alpha \in [0.5, 1]$ is used to control the spreading of generated solution.

In the above stated crossover operation, we denote $\mathbf{X}$ as female parent and $\mathbf{Y}$ as the male parent. As the generated offspring will be near to the female parent, it is therefore considered as an anchor point in the searching process. The diversity of generated solution is governed by the selection of male parent. As in (Deb et al., 2002), this separation of solution also has the function of mutation operator. The next task is to select proper parent.

For choosing the female parent, the uniform fertility selection (UFS) is employed. To apply UFS, first we construct female parent pool by taking $N_f$ number elite solutions and then track number of times each solution is selected from the pool. The less used solution in the female parent pool is chosen as the female parent. UFS focuses on the diversity of the solution generation. As each possible female parent is located at the promising areas, UFS confirms to search these areas throughly.

The negative associative mating (NAS) is used to choose the male parent. The male parent pool consists $N_m$ number of solutions. First, the roulette wheel method is applied to the selected 5 candidate male parents. The Euclidean distance between the selected female parent and each of the possible male parent is calculated. The one with the highest distance is selected as the male parent. NAS also tries to increase the diversity in the offspring.

For balancing the global and local search of GLGA, the female and male differentiation (FMD) process is employed. FMD determines the sexuality of the solution in the current population. This procedure is conducted before UFS and NAS. FMD determines the number of solution in each parent pool, *e.g* $N_f$ *and* $N_m$ *with* $N_f \leq N$ *and* $N_m \leq N$, with either one of them equal to the total number of solutions in the current population $N$. By choosing different configuration of $N_f$ and $N_m$, the GLGA focus on global or local search. As suggested in (García-Martínez et al., 2008), $N_m = N$ and $N_f = N/2$ is used for global searching and $N_m = 100$ and $N_f = 5$ is used for local searching. The rational of this parameter setting is clear from the construction of the algorithm. In the global search phase, the more number of female parents are used to ensure the algorithm covers as much area as possible and the high number of male parents ensure the exploitation. After entering into the local search, the decreased number of female parents would help the algorithm to focus more on the promising area. GLGA is not operated by generation, rather it uses function evaluations as the criterion to shift from global to local search. GLGA-n% indicates that n% of function evaluations are used for global search and rest are used for local search.

To keep the number of solutions in the population consistent, the "replace the worst strategy" is adopted. Each time when an offspring is generated, the fitness value of the offspring is compared to the solution in the current population. If it is better than any of the solution in the current population, the worst one is replaced.

## 4 CMA-ES

The $(\mu, \lambda)$ CMA-ES requires $\lambda$ number of solutions in each generation in order to select the best $\mu$ number of solutions for updating the value in next step. The CMA-ES algorithm updates the solution by sampling from a Gaussian distribution with mean $m$, step-size $\delta$ and covariance estimation $\mathbf{C}$ as follows:

$$s_k^{(g+1)} \sim m^{(g)} + \delta^{(g)} \mathcal{N}(0, \mathbf{C}^{(g)}) \quad \text{for} \quad k = 1, \ldots, \lambda \tag{S11}$$

where $g$ and $k$ are indices for generation and solution respectively, $s_k^{(g)}$ is the $k$th solution in $g$th generation. To find the solution in the next generation, CMA-ES estimates the mean, step-size and covariance by using the solution in the current generation and also considers the history path of the solution evolution. The

mean $m^{(g+1)}$ update is given by:

$$m^{(g+1)} = \sum_{k=1}^{\mu} w_k s_k^{(g)} \tag{S12}$$

After each generation, the solutions are ranked based on their fitness value. The update of mean is carried out by a weighted summation of the $\mu$ best solutions in the current generation. Each weight is user defined and subject to the condition $\sum_{k=1}^{\mu} w_k = 1$.

The step-size $\delta$ is the trade-off between exploration and exploitation of the CMA-ES. Its update requires the evolution history in solution space to calculate evolution path $p_\delta$. The update rule is given by:

$$\delta^{(g+1)} = \delta^{(g)} \exp\left(\beta\left(\frac{||p_\delta||}{\mathrm{E}||\mathcal{N}(0,\mathbf{I})||} - 1\right)\right) \tag{S13}$$

where $||p_\delta||$ is the length of evolution path calculated up to current generation, $\mathrm{E}||\mathcal{N}(0,\mathbf{I})||$ is the length of a normal distribution with the same dimensionality of the solution vector and $\beta$ is a tunable parameter. The expectation is that the solution evolution path denoted by $||p_\delta||$ should distribute similar to a random walk that is characterized by a normal distribution. If the solutions in the consecutive generation tend to move to a similar direction, a bigger $\delta$ is required to explore more area in the solution space. Whereas, if the solutions tend to be in contrary direction, a smaller $\delta$ is used to search in a finer grain.

Finally, the covariance matrix $\mathbf{C}$ needs to be updated. The value of $\mathbf{C}$ is determined by the covariance of the estimated evolution path denoted by $p_c$ and the covariance estimated from the $\mu$ number of solutions in the current generation. The update rule is given as

$$\mathbf{C}^{(g+1)} = (1 - \gamma_1 - \gamma_2)\mathbf{C}^{(g)} + \gamma_1 \mathbf{Cov}(p_c) + \gamma_2 \mathbf{Cov}(s_{1:\mu}) \tag{S14}$$

where **Cov(•)** is the covariance estimation of corresponding variable, $\gamma_1$ and $\gamma_2$ are tunable parameters. In (Hansen et al., 2009), $\mathbf{Cov}(p_c)$ and $\mathbf{Cov}(s_{1:\mu})$ are called rank-one and rank-$\mu$ update respectively. The former term captures the historical information of the solution evolution whereas the latter term contains the solution distribution in the most recent generation. The CMA-ES starts by setting initial value to $s^{(0)}$, $\delta^{(0)}$, $\mathbf{C}^{(0)}$ and updating the solution according to Eq.(S11) to Eq.(S14). The algorithm terminates when certain stopping criterion has been met.

## REFERENCES

Deb, K., Pratap, A., Agarwal, S., and Meyarivan, T. (2002). A fast and elitist multiobjective genetic algorithm: NSGA-II. *IEEE Transactions on Evolutionary Computation* 6, 182–197

García-Martínez, C., Lozano, M., Herrera, F., Molina, D., and Sánchez, A. M. (2008). Global and local real-coded genetic algorithms based on parent-centric crossover operators. *European Journal of Operational Research* 185, 1088–1113.

Hansen, N., Niederberger, A. S. P., Guzzella, L., and Koumoutsakos, P. (2009). A method for handling uncertainty in evolutionary optimization with an application to feedback control of combustion. *IEEE Transactions on Evolutionary Computation* 13, 180–197.

Lotte, F. and Guan, C. (2011). Regularizing common spatial patterns to improve BCI designs: unified theory and new algorithms. *IEEE Transactions on Biomedical Engineering* 58, 355–362.

Samek, W., Vidaurre, C., Müller, K.-R., and Kawanabe, M. (2012). Stationary common spatial patterns for brain-computer interfacing. *Journal of Neural Engineering* 9, 026013.

Veluvolu, K. C., Wang, Y., and Kavuri, S. S. (2012). Adaptive estimation of EEG-rhythms for optimal band identification in BCI. *Journal of Neuroscience Methods* 203, 163–172.

Wang, Y., Veluvolu, K. C., Cho, J.-H., and Defoort, M. (2012a). Adaptive estimation of EEG for subject-specific reactive band identification and improved ERD detection. *Neuroscience letters* 528, 137–142.

Wang, Y., Wang, Y.-T., and Jung, T.-P. (2012b). Translation of EEG spatial filters from resting to motor imagery using independent component analysis. *PloS one* 7, e37665.
